# Direct Sequencing from the Minimal Number of DNA Molecules Needed to Fill a 454 Picotiterplate

**DOI:** 10.1371/journal.pone.0097379

**Published:** 2014-06-02

**Authors:** Mária Džunková, Marc Garcia-Garcerà, Llúcia Martínez-Priego, Giussepe D’Auria, Francesc Calafell, Andrés Moya

**Affiliations:** 1 Área de Genómica y Salud, Fundación para el Fomento de la Investigación Sanitaria y Biomédica de la Comunidad Valenciana (FISABIO-Salud Pública), Valencia, Spain; 2 Instituto Cavanilles de Biodiversidad y Biología Evolutiva, Universitat de València, Valencia, Spain; 3 CIBER en Epidemiología y Salud Pública (CIBEResp), Madrid, Spain; 4 Institut de Biologia Evolutiva, CSIC-Universitat Pompeu Fabra, Barcelona, Spain; University of Milan-Bicocca, Italy

## Abstract

The large amount of DNA needed to prepare a library in next generation sequencing protocols hinders direct sequencing of small DNA samples. This limitation is usually overcome by the enrichment of such samples with whole genome amplification (WGA), mostly by multiple displacement amplification (MDA) based on φ29 polymerase. However, this technique can be biased by the GC content of the sample and is prone to the development of chimeras as well as contamination during enrichment, which contributes to undesired noise during sequence data analysis, and also hampers the proper functional and/or taxonomic assignments. An alternative to MDA is direct DNA sequencing (DS), which represents the theoretical gold standard in genome sequencing. In this work, we explore the possibility of sequencing the genome of *Escherichia coli* from the minimum number of DNA molecules required for pyrosequencing, according to the notion of one-bead-one-molecule. Using an optimized protocol for DS, we constructed a shotgun library containing the minimum number of DNA molecules needed to fill a selected region of a picotiterplate. We gathered most of the reference genome extension with uniform coverage. We compared the DS method with MDA applied to the same amount of starting DNA. As expected, MDA yielded a sparse and biased read distribution, with a very high amount of unassigned and unspecific DNA amplifications. The optimized DS protocol allows unbiased sequencing to be performed from samples with a very small amount of DNA.

## Introduction

Currently, next generation sequencing platforms are continuously improving, in their endeavor to be accurate, fast and cheap, ideally useful to sequence any kind of sample [1]. The main restriction for all platforms is the amount of DNA required for sequencing (e.g. 1 µg of starting material for a rapid library in 454 FLX + technology). However, quite often the amount of DNA available is limited, e.g. biopsies, laser dissection experiments, genomics for non-cultivable microorganisms, single cell genomic experiments, etc. The most commonly used method to increase the initial amount of DNA for sequencing is Multiple Displacement Amplification (MDA) [2], which employs random hexamers to extend genomic fragments by using the isothermal polymerase from the φ29 phage of *Bacillus subtilis* [3]. This reaction was originally designed to amplify circular DNA templates, resulting in 10,000-fold amplification after a few hours [4]. Multiple Displacement Amplification is of special interest in studying selected gene loci by PCR after whole genome amplification [5]–[7]. It has been applied in tumor genetics, single cell microbial genomics and viral genomics [8]–[12].

However, it is also widely known that the MDA reaction may produce genome coverage bias [4] mainly caused by different inter-primer distances [13], resulting in a low coverage or even unamplified regions. In addition, regions of high GC content could lead to amplification biases [14]. Finally, the low specificity of the random hexamers together with an amplification temperature of 30°C make the MDA reaction prone to amplifying template-free hexamer concatenations, to be contaminated by alien sequences, and to the formation of chimeric sequences [15].

To overcome the limitations of MDA, a number of protocol improvements [16]–[19] or novel bioinformatics approaches [20], [21] have been developed.

For 454 pyrosequencing, 1 µg of DNA is required for library preparation yielding picograms of the prepared library [22]. Most of that library is spent for the titration of the emulsion PCR (emPCR), which involves mixing single-stranded library templates with DNA-capturing sepharose beads in an oil emulsion, expecting thus to capture single sequences. The successful quantification of the number of molecules is critical in order to reduce the amount of starting material needed to sequence a DNA sample. Meyer and collaborators [23] used qPCR (quantitative polymerase chain reaction) to quantify the prepared library and calculate the exact template/bead ratio avoiding emPCR titration steps, consequently reducing the amount of starting DNA needed to sequence a given sample. Zheng and collaborators [24] described an alternative method to prepare 454 libraries, wher! e the A and B standard 454 adaptors are replaced by custom 454 adaptors in “Y” form and where the exact number of amplifiable molecules is subsequently quantified by qPCR with MGB (Minor Groove Binder) Taqman probes. These probes allow quantification of small amounts of DNA down to a few zeptograms (10^−21^ grams), even below the minimum amount needed for proper sequencing [25]. These improvements have opened the way to Direct Sequencing (DS) starting from very small amounts of DNA and skipping the whole genome amplification step.

The objective of this work is to obtain a 454 shotgun library for DS starting from the amount of DNA needed to reach exactly the minimal number of molecules required to fill the target picotiterplate (PTP) region. We replaced the steps in which DNA is lost in the standard 454 protocol by more sparing alternatives and quantified these minimal libraries with the qPCR assay designed by Zheng and collaborators [26]. To assess whether DS can be proposed as an alternative to MDA we compared the sequencing results obtained using both methods.

## Results

### Library Preparation and Sequencing Results

**Figure 1:**
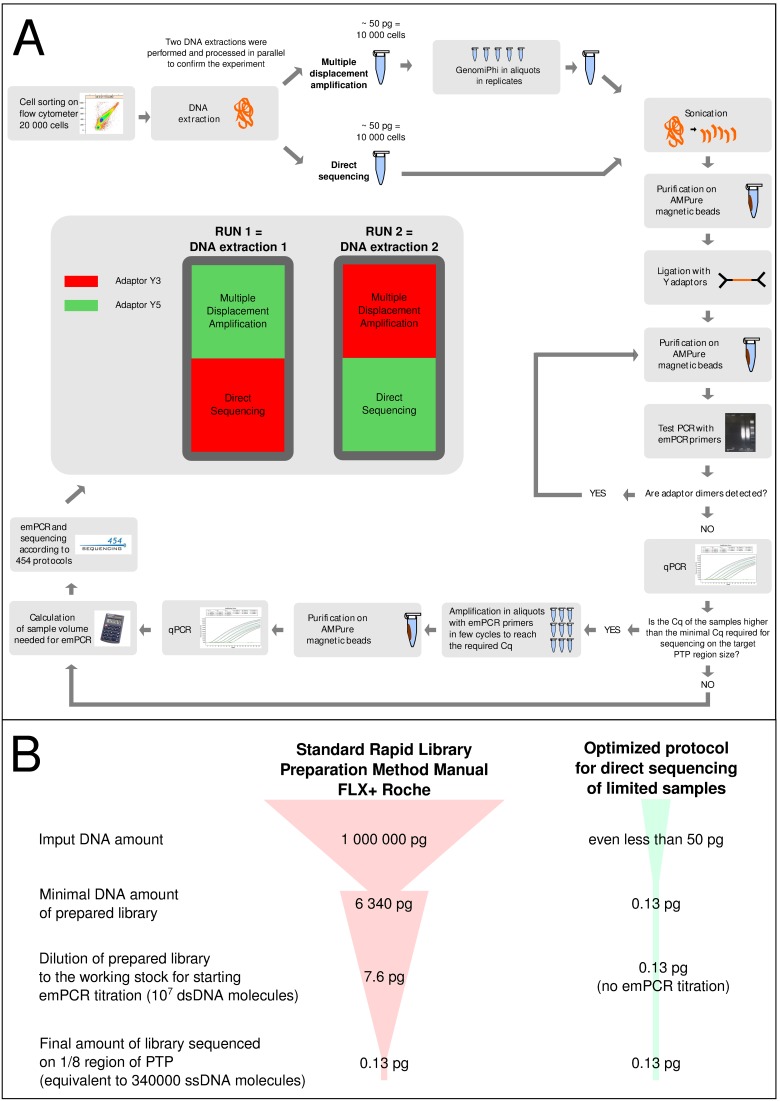
Flowchart of the minimal library preparation protocol. Panel A: The experimental work started with cell sorting, where 20,000 cells were separated in two replicates to confirm the whole experiment. The DNA from 20,000 cells was extracted and split into halves, where one half was amplified with GenomiPhi (MDA) and a second half was processed without whole genome amplification (DS). The shotgun libraries were prepared with the same alternative protocol for the both MDA and DSsamples. Library quality control points were the test PCR with emPCR primers to prove the removal of self-ligated adaptors and the library concentration checking with qPCR. The MDAsample and DSsample with different MIDs in two repetitions were combined into two sequencing runs as sho! wn in the scheme. Panel B: DNA amount requirements in the standard Rapid Library Preparation Method Manual GS FLX+ Series – XL+ (May 2011) compared with the amounts actually needed for sequencing on a selected PTP region. The minimal amount of prepared library required for proceeding to emPCR step in the standard 454 protocol may lose 99% of input DNA during the library preparation step. Then, this amount is diluted to a working stock of 10–7 molecules, defined as the best starting point to perform the emPCR titration step. However, if the exact number of molecules is quantified with qPCR, the emPCR titration step can be omitted, so actually only 0.13 pg of prepared library are needed for sequencing on 1/8 region of PTP (equivalent to 340,000 ssDNA molecules). This allows to use an alternative shotgun protocol where the DNA losses are reduced.

We used flow cytometry and cell sorting to obtain aliquots of 20000 *E. coli* cells. Extracted DNA was divided in two sub-samples containing DNA equivalent to 10000 cells each. Previous setting-up experiments showed that about 10000 bacterial cells, of genome size close to the one of *E. coli,* was the minimal number required to obtain enough DNA to fill 1/8 of a PTP plate (data not shown): the first sub-sample was not amplified and came directly from DNA extraction to library preparation (DSsample) while the second underwent MDA-WGA (MDAsample). Both sub-samples were then processed by optimized 454 library preparation protocol (Figure S1), using two adaptors (Y3 and Y5) with different MID tagging. To calculate the exact number of DNA molecules in the library, the samples were quantified by MGB-TaqMan probe qPCR [26]. The whole process was performed in two replicates to confirm the experiment and is schematically presented in Figure 1.

Quantification of DSsample-Y3 and DSsample-Y5 libraries resulted in 414,443 and 41,043 ssDNA (single strand DNA) molecules respectively. Given that DSsample-Y5 did not contain the required number of molecules as planned for sequencing on a half of 1/8 PTP (170,000 molecules, the second half of PTP was filled with MDAsample), both DS libraries were enriched by 4 PCR cycles using emPCR primers to amplify the sample just sufficiently to reach the minimal required number of molecules. The samples were then quantified again to test whether the amount of DNA was sufficient, then 5,773,461 and 384,561 ssDNA molecules of DSsample-Y3 and DSsample-Y5 were obtained, respectively (see Table S1). MDAsample libraries yielded more DNA and were diluted down to the same concentration as DSsample enriched libraries and pooled together DSsample-Y3 with MDAsample-Y5 (run1) and DSsample-Y5 with MDAsample-Y3 (run2, see Figure 1).

Sequence quality assessment of the run1 had an output of 63,305 sequences (average quality score 36.05). However, the run2 resulted only in 5,762 reads passing quality assessment filters. Still, further analyses showed equivalence for both runs, despite the difference in Mbp obtained. A sequencing overview after quality assessment is shown in Table 1. Finally, both datasets belonging to DSsample and MDAsample experiment were joined resulting in 20,927 and 48,140 reads respectively.

**Table 1:** Sequencing results.

		Direct sequencing	Multiple displacement amplification
**Total number of reads**	Run1	16758	46547
	Run2	4169	1593
**Total Mbases**	Run1	3853532	12891425
	Run2	582005	253376
**Average read length**	Run1	229.95±137.26	276.96±165.51
	Run2	139.60±89.85	159.06±111.82
**Average read quality**	Run1	36.18±3.48	35.91±3.52
	Run2	31.12±3.41	30.84±3.41
**GC content (%)**	Run1	48.94	46.05
	Run2	48.54	46.15
**Theoretical ** ***E. coli*** ** coverage of all** **processed reads**	Run1 + Run2	0.96	2.83
**Theoretical ** ***E. coli*** ** coverage** **of all reads mapped to ** ***E. coli***	Run1 + Run2	0.77	0.07
**Actual obtained ** ***E. coli*** ** coverage**	Run1 + Run2	0.76	0.05

The table shows the number of total reads, total bases, mean and median read length and mean GC content (%) of processed sequences. The theoretical *E. coli* coverage was calculated from the total obtained Mbp. In fact, the actual coverage was lower than expected, especially in the case of MDAsample.

We observed a significant decrease in GC content in the MDAsample (46.10%) compared to the DSsample (48.74%, t-test, p-value = 0.0021).

### *E. coli* Genome Mapping

**Figure 2:**
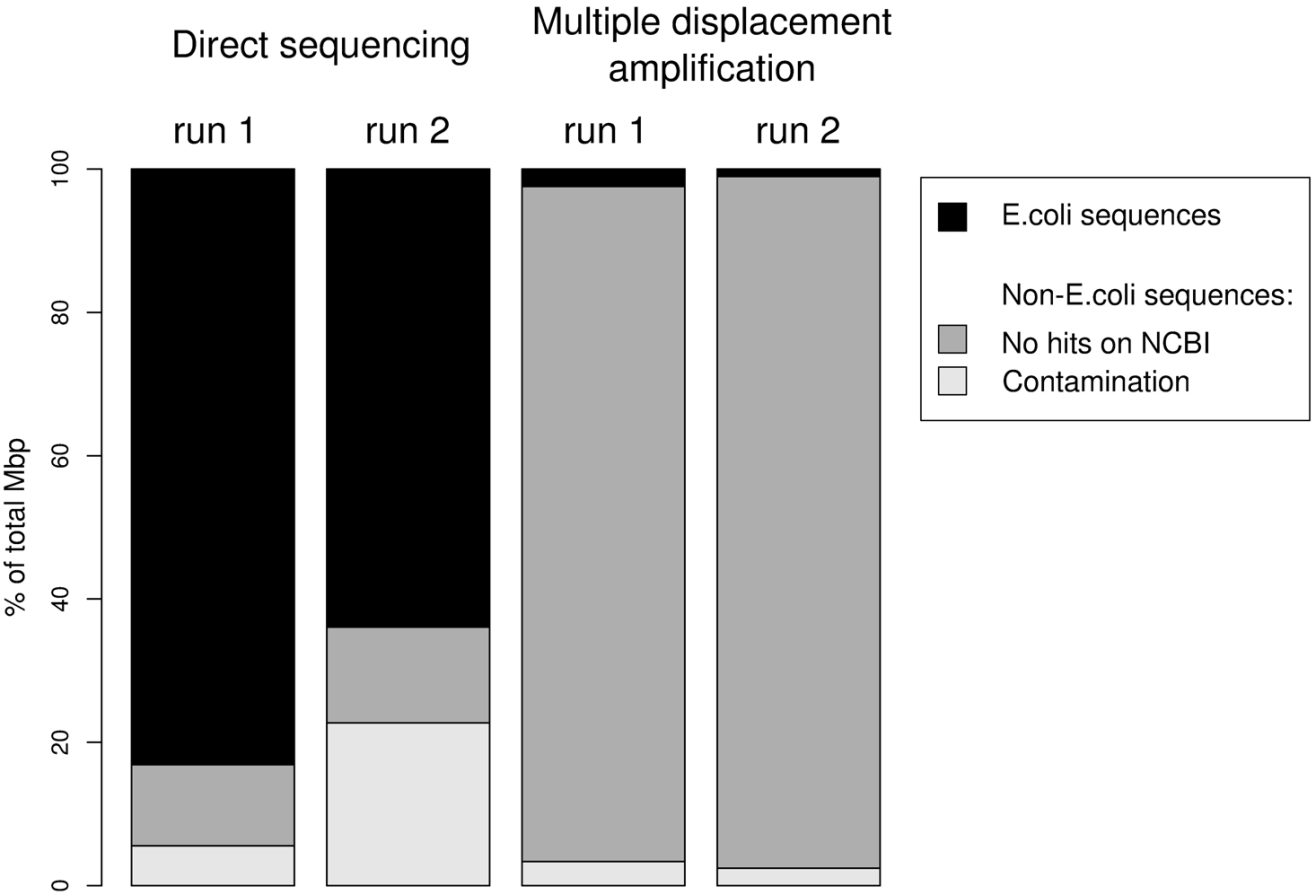
Results of *E. coli* genome mapping and blast to NCBI database. Proportions (in %) of Mbp mapped by SSAHA2 to *E. coli* genome are shown for MDA and DS sequences, separately for each sequencing run. It can be observed that the percentage of mapped DS reads were significantly higher than the MDA reads. The reads that were not mapped to *E. coli* were analyzed by blast in “nr” database. However, most reads remained unidentified, especially in the case of MDA.

**Figure 3:**
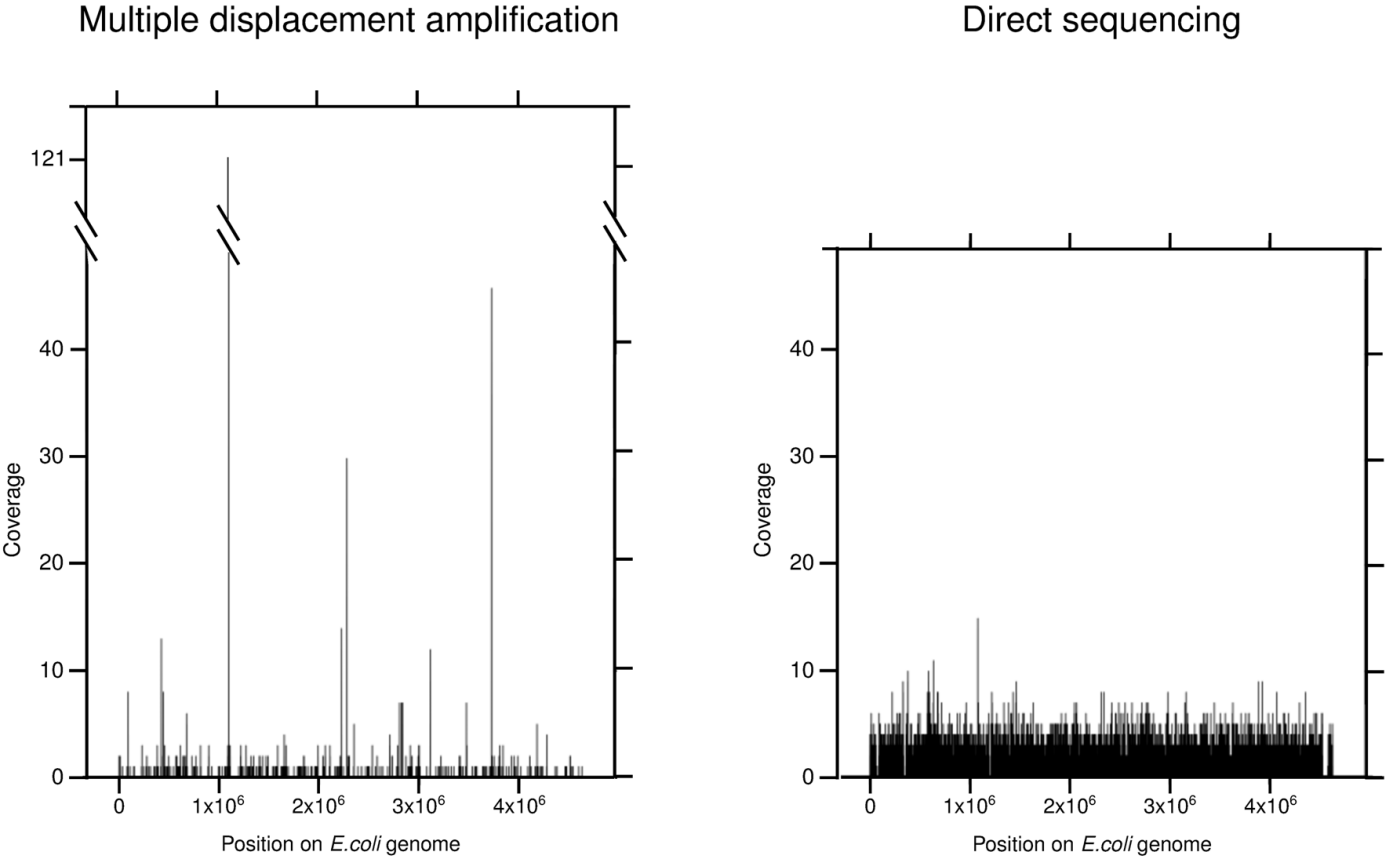
Distribution of coverage throughout the *E. coli* genome. The comparison of the genome coverage obtained by MDA and DS methods. The genome coverage of MDA reads was characterized by unequal distribution with many gaps and several areas with extremely high coverage (up to 121 x), while the highest coverage obtained by DS was only 15 x and it was better distributed throughout the whole genome.

Although we obtained three times more sequences in the MDAsample than in the DSsample, both methodologies were theoretically sufficient to cover the whole *E. coli* genome (Table 1). However, the DSsample covered a greater part of the reference genome (47.43%) than the MDAsample (2.45%). Moreover, only 2.10% of the sequences of the MDAsample matched the *E. coli* genome, whereas this figure was 80.59% for DSsample (Figure 2). Finally, the genome coverage associated with the MDAsample was characterized by peaks of overrepresented regions up to 121 X with an average coverage of 0.05 X; by contrast, the DSsample showed a maximum coverage of 15 X but with a uniform distribution with an average value of 0.76 X, fifteen times higher than the average coverage of the MDAsample (see Figure 3). The coverage distributions obtained with both methods were significantly different (one way Kruskal-Wallis test, p-value = 0.0017).

### Analysis of Unassigned Reads

1,460 reads (6.98% of total Mbp) from the DSsample and 1,423 reads (2.96% of total Mbp) of MDAsample datasets did not map to the *E. coli* genome but to other species in “nr” database. Approximately one third of these sequences were identified as human and the rest were assigned to other bacteria, mainly *Proteobacteria*. Moreover, in both samples we also found reads that were not assignable to any organism present in the “nr” database. More precisely, 94.2 and 96.54% in the MDAsample and 11.35 and 13.38% in the DSsample were present for total processed Mbp in their respective runs 1 and 2 (Figure 2). It is worth noticing that the unclassified reads showed the same quality and length ranges as the *E. coli*-mapped reads.

Regarding GC content, it was lower in the unassigned reads of MDAsample (46.1%) than the unassigned reads of DSsample (49.47%) (Figure S2). When unassigned reads from MDAsample were grouped by similarity, we observed an increasing size of clusters, along with a decrease in similarity stringency (from 100% similarity down to 70%). On the contrary, the similarity range of unassigned reads of DSsample was not affected by cluster size (Figure S3).

**Figure 4:**
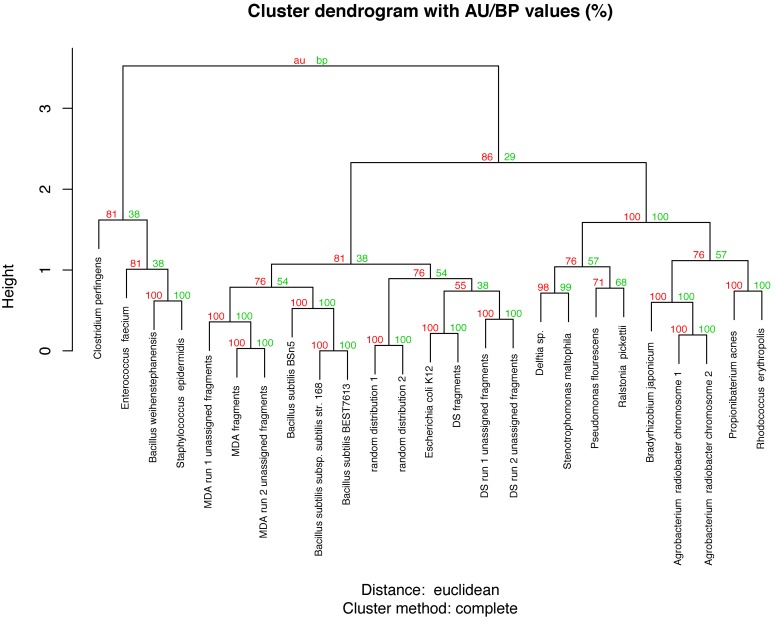
Clustering analysis of the k-mer abundance distribution. Comparison of the relative abundances of 6-mer in the different datasets using hierarchical clustering. As observed, the most likely conformation shows aggregation of *E. coli* with DS methodology, while *B. subtilis* is associated with MDA.

In order to explain the origin of the unassigned reads, we explored the distributions of hexamers in the two method datasets. Unassigned read hexamer distributions of MDAsample as well as DSsample were significantly different from the normal distribution of the artificial genome based on an average purine-pyrimidine ratio of 0.5 (Cramer von Mises test, p-values = 2.99×10^−11^ and 6.69×10^−8^, respectively, Figure S4). We observed that the calculated hexamer distribution of reads from other selected genomes from public repositories displayed a gamma distribution of hexamers similar to the one we observed in both our methods (Cramer von Mises test, p-values ranging from 0.16 to 0.51, depending on the genome, Figure S5). Hierarchical clustering with bootstrap reconciliation of hexamer relative abundance profiles showed that all reads coming from the DSsample were adjacent to the *E. coli* hexamer profiles. Unassigned reads clustered together on the most likely conformation clustering. However, it was not statistically supported by the bootstrap analysis (bootstrap support = 55%). On the other hand, the distribution of MDAsample unassigned reads was statistically far from *E. coli* distribution, but close to *Bacillus subtilis* genomes (Bootstrap support = 100%, Figure 4).

## Discussion

In the standard FLX+ Rapid Shotgun Library preparation protocol, 1 µg of input DNA is needed, while the minimal required output of ∼700 bp DNA fragments resulting from this protocol is 8.4×10^9^ dsDNA molecules (6,340 pg), losing up to 99.36% of the initial amount of DNA. Furthermore, the resulting amount of the library is diluted up to 10^7^ molecules, which is supposed to be the correct starting concentration for emPCR titration [22]. This requirement results in an insurmountable obstacle for limited or rare samples. To overcome this drawback, the most widely used method has been isothermal MDA. However, MDA entails a number of serious problems, which have been reported previously.

Several authors have already suggested that next-generation sequencing library preparation protocols can be started with a lower amount of DNA than that required by the standard protocol. They stated that the true limiting factor, in the case of 454 sequencing, is to reach the number of enriched beads required by the platform [23], [24], [27]–[29]. In this work, we demonstrate that it is possible to prepare a 454 shotgun library for direct sequencing, starting with the amount of DNA needed to reach the exact minimal number of molecules required to fill the target picotiterplate (PTP) region (e.g. 340,000 enriched beads for 1/8th PTP plate, equivalent to 0.13 pg of 700 bp fragments). To asses this hypothesis, we reduced sample losses by modifying the standard Roche protocols using thriftier alternatives. It is well known that nebulization is responsible of DNA losses during the library preparation process. Rather than fragmenting the sample in a nebulizer, we sonicated it in the same tube in which DNA extraction was performed, thus also avoiding losses due to sample transfer. Small fragments were removed exclusively with AMPure magnetic beads. These changes have been already reported as good alternatives of the standard protocol and they can be used at a large scale [29].

Assessing the minimum number of enriched beads/molecules required by the 454 platform calls for highly sensitive quantitative methods. Minor Groove Binding (MGB) and Locked Nucleic Acid (LNA) probes are surely some of the most sensitive DNA quantification systems [30]. The Roche standard rapid shotgun library method uses a Y-adaptor with a fluorochrome, which quantifies the number of correctly ligated molecules by a fluorometer. However, its sensitivity is much lower than that of MGB probes. Roche standard adaptors interfere with MGB probes by using similar absorption and emission wavelengths. To avoid this, two alternatives may work: using a MGB label that does not interfere with Roche Y adaptors, or synthesizing Y adaptors without the fluorochrome [26]. Thus, we used Y adaptors with MGB-Taqman probes designed by Zheng and collaborators, enabling us to quantify the exact number of amplifiable molecules of these minimal libraries by qPCR [26]. Once the exact number of amplifiable molecules is calculated, the user can proceed directly to proper emPCR without the need to perform the previous emPCR titration step, saving further DNA [23], [26], [29].

Zheng and collaborators [24] started with 1 ng of size-selected nebulized fragments, ligated custom Y adaptors and generated a library that was sufficient for 10 Titanium runs. In this work, we started with an even lower DNA concentration, without diluting the original DNA sample, and preparing a sufficient minimal library able to be sequenced in one Titanium run (1/8 region of PTP). Due to the fact that DNA losses during extraction and small fragment removal cannot be avoided, the amount of size-selected sonicated fragments in our work was definitely lower than 50 pg (theoretically, the DNA content of 10,000 *E. coli* cells). To design sequencing experiments of limited samples, we must always have sufficient sequence coverage and some sample losses in DNA extraction and library construction. Eve! n if no DNA losses took place, a typical bacterial cell containing 6–16 fg of DNA would never provide a sufficient number of molecules for high-throughput sequencing [25]. To this end, Table S1 helps users choose the appropriate PTP region size for sequencing quantified minimal libraries. If the number of molecules required to fill the smallest PTP region (1/8th is the smallest PTP subdivision most widely used) is not obtained, then amplification by PCR with emPCR primers with a low number of cycles could be performed.

In this work, the performance of direct sequencing (DS) was compared with the widely applied multiple displacement amplification (MDA) approach used for samples with small amounts of DNA. Direct sequencing provided homogeneous genome coverage throughout most of the genome. By contrast, MDAsample generated regions with a genome coverage as high as 121 X, leaving almost all of it (97.55%) uncovered. This observation is in accordance with reports by other authors using MDA, who estimated that more than 40% of the sequence could be missing, and that the heterogeneous coverage distribution in MDA frequently leads to failure, without being able to hone in on the target genome by further sequencing efforts either [11], [16], [21], [31]. In spite of these shortcomings, MDA has been used to discover novel genes or complex regulatory systems [9], [11], [32].

Another problematic issue entailed by working with so little starting sample is obviously contamination with foreign DNA. In DS experiments, we obtained sequences that matched ‘nr’ database but did not belong to the *E. coli* genome, moreover, the same kinds of contamination were observed in parallel with the MDA experiment. We think that flow cytometry might be the source of these non-target sequences. Although the low volume surrounding each sorted single cell reduces the contamination of extracellular DNA [33], it is very difficult to completely avoid contamination during cell sorting [16]. Flow cytometry could also be the source of chimera formation, as the sodium hypochloride used for cell sorter cleaning can break DNA by depurination [34]. This is especially true when MDA is used subsequently, because even short DNA remaining from previous equipment usage may constitute a DNA source for chimera construction.

In metagenomic experiments, it is common to retrieve a variable percentage of sequences unknown to public repositories, depending on the environment [35]. However, single genome targeted sequencing can also produce a variable number of unknown reads [36]–[38]. In this work, although the cells used for DNA extraction in both approaches (DS and MDA) were obtained by flow cytometry sorting, the number of unassigned sequences was considerably lower in DSsample (12.84%) than in that obtained by MDAsample (up to 94.24%). The commercially available MDA reagents have frequently been reported as being contaminated by unwanted DNA. Several authors dealing with MDA contamination concluded that contamination did not come from human DNA or other target genomes, but could originate from hexamer concatenation or from the enzyme preparation process, including host bacteria *Bacillus subtilis* [18], [20], [31], [39]–[41]. Theoretically, samples from the same genomic origin should present equivalent or similar hexamer content. This method was previously used to compare metagenomic sources, allowing the discrimination of samples with potential contamination [42]. Our results support these conclusions, since we observed that MDA unranked reads were close to the *B. subtilis* genome in hexamer composition.

## Conclusions

In this work we used direct genome sequencing (DS) on a 454 Titanium platform starting from minimal amounts of input DNA without previous whole genome amplification. This approach could replace MDA in many genome sequencing projects, even in studying previously uncharacterized organisms. Direct sequencing provides unbiased, reliable and reproducible genetic information from any sample with a minimum amount of starting material. In contrast to MDA, which is widely applied to projects dealing with limited amounts of DNA, DS provides a homogeneous distribution of reads mapped to a reference genome, avoiding low efficiency, chimera formation and amplification problems previously described in MDA. We propose that DS is a candidate to replace MDA in most “omic” projects including RNAseq experiment, given that its sequencing efficiency reduces the cost of library preparation and maximizes the gathering of genetic information.

## Methods

### Strains and Media

The *Escherichia coli* strain K12 was cultured overnight (O/N) in liquid Lysogeny Broth medium at 37°C. The culture was pelleted by centrifugation at 805 rcf for 4 min at 4°C, and washed twice in cool physiological solution (NaCl 0.9%). Cells were immediately fixed adding formaldehyde 3.7%, and incubated O/N at 4°C. Fixed cells were washed twice to remove the remaining of formaldehyde and resuspended in physiological solution to reach 0.1 OD 600 (optical density).

### Flow Cytometry Separation of 10,000 Cells

*E. coli* cells were stained with SYTO62 DNA staining (Invitrogen, Paisley PA4 9RF, UK, #S11344) according to manufacturer’s instructions. Flow cytometry sorting was performed using a MoFloTM XDP cell sorter (Beckman-Coulter, Pasadena CA). Wavelength emission was set at 635 nm, and absorption at 670 nm, to detect signal from the *E. coli* DNA stain. Gates were set using the side-scatter vs. fluorescent signal to separate the cells. Sorted cells were placed in 1.5 mL sterile tubes containing physiologic solution to reach 20,000 cells.

### DNA Extraction

Two DNA extractions from two aliquots of 20,000 sorted cells were carried out in order to replicate the experiment. All the chemicals used were previously sterilized by autoclave plus filtration through 0.2 µm-pore sterile filters. DNA was extracted according to the protocol by Ausubel and collaborators [43] in sterile conditions. DNA was resuspended in 20 µL nuclease- free water and divided in two sub-samples (DSsample and MDAsample).

### Multiple Displacement Amplification (MDA)

MDA was performed with the GenomiPhi V2 amplification kit (GE Healthcare Waukesha, WI, #25-6600-30) following manufacturer’s instructions with incubation at 30°C for 2.5 hours. In order to reduce amplification bias, we performed the amplification in 5 replicates using 2 µL of extracted DNA per tube, and finally pooled back after the reaction finished. After this step, this DNA sample (MDAsample) was processed in parallel with its unamplified counterpart (DSsample).

### Shotgun 454 Library Preparation

Figure S1 shows the changes to standard Roche FLX+ Rapid Library preparation protocol proposed here. Sample volumes were brought up to 100 µL. DNA was sheared using a Raypa UCI-50 sonicator which allows working with closed tubes, thus avoiding contamination. Sonication was carried out at 2°C for 3 minutes at maximum intensity, obtaining a fragment distribution of 200–1000 bp. DNA fragments shorter than 400 bp were removed by Agencourt AMPure Beads XP (Beckman-Coulter, #A63881). According to the bead calibration, we added 1.2 volumes of AMPure beads to our sample (v/v). The mixture was incubated for 3 min and placed on a magnetic particle concentrator (Invitrogen, #123-21D) for 3 min to let the beads pellet. The supernatant was discarded and the bead pellet was washed twice with 70% ethanol. The pellet was dried and finally resuspended in 12 µL of water.

The 454 adaptor ligation was performed according to the protocol proposed by Zheng and collaborators [26]. The 12 µL of DNA from previous step was added to a blunt-end mixture containing 1 µL of dNTPs 25 mM each (Fermentas, Thermo-Fischer, Waltham, MA, #R0181), 2.5 µL of ligation buffer (New England Biolabs, Ipswich, MA, #M0202S), 2.5 µL of ATP (Agilent, Santa Clara, CA #200340-81), 2 µL of quick blunting enzyme mix (NEB, #E1201L) and 0.5 µL of Klenow Fragment 3′ 5′ exo- (NEB, #M0212S). The mixture was incubated in a thermocycler (Eppendorf, Hamburg, Germany) for 15 min at 12°C followed by 15 min at 72°C. After this incubation, the solution was ice-cooled.

Two Y adaptors (synthesized by Sigma-Aldrich, St. Louis, MO) with two different multiplex identifiers (MIDs, Y3 and Y5) were prepared according to the Zheng and collaborators [24] protocol in order to tag the DSsample and the MDAsample for the further sequencing step. A biological replicate (including DNA extraction, MID tagging, and sequencing) was performed. Since MID-adaptor hybridization might result in an experimental bias, the adaptors were exchanged (Figure 1, panel A). 1 µL of each Y adaptor (100 µM for initial concentration) was added to the repaired DNA from previous step. 1 µL of T4 DNA ligase (NEB #M0202S) was added to the mixture. The whole solution was incubated at 12°C O/N. After incubation, possible self-ligated adaptors were removed with AMPure bead purification as previously described. Purified samples were then resuspended in 50 µL of water.

### Library Quality Control

In order to confirm adaptor ligation and correct library fragment size after purification, we prepared a test PCR using as primers the A and B 454 adaptor sequences (emPCR-F 5′- CCATCTCATCCCTGCGTGTC-3′, empCR-R: 5′-CCTATCCCCTGTGTGCCTTG-3′, synthesized by Isogen Life-Science (De Meern, The Netherlands). In the PCR reaction, 1 µL of sample was mixed with GoTaq Green polymerase Mix 2x (Promega, Fitchburg, WI, #M7112) and 1 µL of each emPCR primer (10 µM initial concentration), using the following conditions: the initial denaturation step at 94°C for 2 min, followed by 25 cycles of 94°C for 2 sec, 60°C for 60 sec, and 72°C for 60 sec, and a final extension at 72°C for 8 min. The PCR product was visualized in a 0.8% agarose gel performed under standard conditions. Self-ligated adaptors were observed as a band around the 100 bp region. In this case, we repeated the purification step until the band became undetectable (Figure S6).

### Quantitative PCR

Quantitative PCR was performed on a Roche LightCycler LC480 II, with a MGB-TaqMan probe, according to Zheng and collaborators [26]. Each test was repeated three times. For each reaction, the mix was prepared as follows: 10 µL of Kapa Probe Fast Universal 2x qPCR master mix (Kapa Biosystems, Woburn, MA, #KK4701) with 1.4 µL of each emPCR primers, 1.2 µL of MGB-TaqMan probe 10 µM and 1 µL of DNA sample. The reaction was finally adjusted to 20 µL with nuclease-free water. To calculate the exact number of molecules, a standard curve was prepared using an amplification product of known length (202 bp) and known concentration which contained the same adaptors used for 454 sequencing. Serial dilutions 1∶10 of the standard were prepared and amplified with the same qPCR protocol. Quantitative PCR was performed as follows: the first denaturation step at 94°C for 10 minutes was followed by 40 cycles of 95°C for 30 sec, 60°C for 15 sec and 68°C for one minute, allowing the longer reads to be extended.

MGB-TaqMan probe qPCR allowed us to calculate the exact number of molecules, independently of the fragment length. A standard curve was used to derive the Cq (quantification cycle value) vs log (number of molecules) linear equation. We then used that equation to calculate the exact number of molecules per microliter in our samples. The calculation for the exact library quantification is reported in Table S1. Samples that did not reach the minimum number of molecules required for sequencing on target PTP region were further enriched with PCR using the emPCR primers applying the same PCR conditions as for library quality control previously described. The calculation of the number of cycles required to reach the minimal number of molecules for filling the target PTP plate are shown in Table S1. This enrichment was performed separately in 20 tubes with 2 µL of starting material to avoid possible PCR bias.

### Emulsion PCR and Sequencing

MDAsample and DSsample were prepared using different MIDs by combining the two approaches on the same 1/8 PTP plate. We calculated the sample volume needed to obtain 340,000 beads (170,000 molecules per one MID) with 5–15% enrichment as recommended by Roche. The emPCR was prepared with the Small Volume emPCR kit (Roche Applied-Science. Penzberg, Germany, #05618444001) and later sequenced using a GS FLX Titanium Sequencing XLR70 Kit (Roche Applied Science, #5233526001).

### Sequence Processing

The sequences obtained were filtered and trimmed by quality and checked for the presence of Y adaptors in the 3′ end using Blast [44] with a match e-value below 10^−3^. Blast results were parsed to determine the adaptor coordinates. Those coordinates were used for sequence trimming with the Biostrings v.2.11 R package [45], [46]. Low complexity reads were removed from the analysis using an R homemade script including the functions from the Short Read [47] and Biostrings and Entropy [48] packages, respectively. The bioinformatics downstream analysis pipeline is shown in Figure S7.

### Genome Mapping and Data Analysis

Reads from both experiments were mapped against the genome of *E. coli* K12 (gi: 49175990) using SSAHA 2.5.4 [49] (word size 13, minimum length for cross match 10, word size for cross_match = 10, number of kmer 1). Coverage was obtained by applying the R packages Rsamtools [50], ShortRead and Chipseq [51]. The distribution of coverage differences between both methodologies was checked using Cramer von Mises test with the CvM2SL2Test R package [52]. We also tested for normality of the coverage and the differences between coverage distributions among both sequencing methods by subsampling the datasets 100 times 1000 reads each.

Reads that did not match *E. coli* were aligned using NCBI-blast against the “nr” database using the Megablast algorithm. The presence of read clusters (duplicated reads) was explored using CD-HIT software on a range of stringency values [53]. In order to examine the origin of reads not matching any “nr” database entry, a hexamer distribution analysis was carried out by again applying the Cramer von Mises test [52]. Hexamer distribution of unassigned reads generated by both sequencing methods was compared with the hexamer distributions of complete genomes chosen from best matches of non-*E. coli* reads. As a null distribution, we constructed an artificial genome based on an average purine-pyrimidine ratio of 0.5, and calculated the hexamer distribution for that genome. Hexamer relative abundance statistics was estimated by applying the R package Vegan [54]. To assess similarities between the different groups, ANOVA was performed using the correlation eigenvalues and the different theoretical clusters. The robustness of hierarchical grouping among different groups was measured with a bootstrap analysis with 1000 generations. Hierarchical clustering was performed using the R packages hclust and pvclust [55].

### Data Access

Sequences were deposited in EMBL-EBI Sequence Read Archive (SRA) with study number ERP003418 (http://www.ebi.ac.uk/ena/data/view/PRJEB4158).

## Supporting Information

Figure S1 **Steps in standard Rapid Library Preparation GS FLX+ Series – XL+ (May 2011) compared with optimized protocol used in this work.** The main changes include sonication for DNA fragmentation (not nebulization), small fragments were removed exclusively with AMPure beads, and alternative chemicals were used for library preparation. The library quality was checked with a test PCR with emPCR primers (instead of using Agilent analyzer) and quantified with qPCR instead of a fluorometer.
(PDF)

Figure S2 Panel A: Comparison of read length, read complexity, sequence quality and GC content among total reads, *E. coli* mapped reads and unassigned reads of both approaches (MDA and DS) and both runs. The results in the Table indicate that run 2 performed worse than run 1, but both runs confirmed the same results. The difference between MDA and DS datasets can be observed here only by lower GC content in MDAsample. Panel B: Distribution of read lengths. Distribution of length of total processed reads, reads mapped to *E. coli* and unclassified reads are compared between both runs of MDA and DS. No differences were found.
(PDF)

Figure S3 **Clustering of unclassified reads with CD-HIT.** The sequences that were not assigned to any species were clustered on different sequence identity levels (from 99 to 75%) allowing us to cluster sequences with 80% length of the cluster. This figure shows the decreasing number of clusters (the increasing size of clusters) by decreasing stringency. Both runs (run 1+ run 2) were processed together for each method (MDA and DS). MDAsample was characterized by abrupt clustering, which demonstrates that the MDAsample reads originated by amplification; however, a high number of clusters was still present at 75% identity level, indicating their uniqueness.
(PDF)

Figure S4 **Correspondence analysis of the k-mer relative abundances.** Comparison of *E. coli*, *B. subtilis* k-mer distributions versus a random k-mer distribution. The taxonomic allocation of the unassigned reads in both methods was obtained by using the eigenvalue coordinates for the k-mer relative abundances for each dataset.
(PDF)

Figure S5 **Amplification of the k-mer abundance space spectra on the Figure S4, by including phylogenetically distant genomes on the correspondence analysis.** As a result we observe a better aggregation of each methodology dataset to its respective expected phylogenetic source, as observed with other statistical methods.
(PDF)

Figure S6 **Quality control of minimal 454 libraries after purification step.** This figure shows quality control of libraries DSsample-Y3 and DSsample-Y5 after the 2nd and 4th purification steps. If the concentration of sample DNA fragments is very low, the adaptor:fragment ratio is high and therefore repeated removal of self-ligated adaptors by AMPure beads must be performed. After the first purification, usually only self-ligated adaptors are visible, because they are shorter than the library and therefore amplify better. After each of these purification steps, the amount of self-ligated adaptors is reduced and the library fragments become more visible.
(PDF)

Figure S7 **The scheme showing bioinformatics analysis pipeline used in this work.**
(PDF)

Table S1 **Calculation of number of molecules needed for minimal library preparation.** The file shows how the number of molecules contained in the library was calculated in this work. Users can replace the green cells with their own measured values.
(XLS)

## References

[1] Wolinsky H (2007) The thousand-dollar genome. genetic brinkmanship or personalized medicine? EMBO Rep 8: 900–903. 17906669 10.1038/sj.embor.7401070 PMC2002559

[2] Binga EK, Lasken RS, Neufeld JD (2008) Something from (almost) nothing: the impact of multiple displacement amplification on microbial ecology. ISME J 2: 233–241. 18256705 10.1038/ismej.2008.10

[3] Vlček Č, Pačes V (1986) Nucleotide sequence of the late region of bacillus phage phi 29 completes the 19285-bp sequence of phi 29 genome. Comparison with the homologous sequence of phage PZA. Gene 46: 215–225. 3803926 10.1016/0378-1119(86)90406-3

[4] Dean FB, Nelson JR, Giesler TL, Lasken RS (2001) Rapid amplification of plasmid and phage DNA using Phi29 DNA polymerase and multiply-primed rolling circle amplification. Genome Res 11: 1095–1099. 11381035 10.1101/gr.180501 PMC311129

[5] Paez JG, Lin M, Beroukhim R, Lee JC, Zhao X, et al (2004) Genome coverage and sequence fidelity of phi 29 polymerase-based multiple strand displacement whole genome amplification. Nucleic Acids Res 32: e71. 15150323 10.1093/nar/gnh069 PMC419624

[6] Dean FB, Hosono S, Fang L, Wu X, Faruqi AF, et al (2002) Comprehensive human 25genome amplification using multiple displacement amplification. Proc Natl Acad Sci U S A 99: 5261–5266. 11959976 10.1073/pnas.082089499 PMC122757

[7] Raghunathan A, Ferguson HR, Bornarth CJ, Song W, Driscoll M, et al (2005) Genomic DNA amplification from a single bacterium. Appl Environ Microb 71: 3342–3347. 10.1128/AEM.71.6.3342-3347.2005 PMC1151817 15933038

[8] Yoon HS, Price DC, Stepanauskas R, Rajah VD, Sieracki ME, et al (2011) Single-Cell genomics reveals organismal interactions in uncultivated marine protists. Science 332: 714–717. 21551060 10.1126/science.1203163

[9] Podar M, Abulencia CB, Walcher M, Hutchison D, Zengler K, et al (2007) Targeted access to the genomes of low-abundance organisms in complex microbial communities. Appl Environ Microb 73: 3205–3214. 10.1128/AEM.02985-06 PMC1907129 17369337

[10] Allen LZ, Ishoey T, Novotny MA, McLean JS, Lasken RS, et al (2011) Single virus genomics: A new tool for virus discovery. PLoS ONE 6: e17722. 21436882 10.1371/journal.pone.0017722 PMC3059205

[11] Marcy Y, Ouverney C, Bik EM, Lösekann T, Ivanova N, et al (2007) Dissecting biological “dark matter” with single-cell genetic analysis of rare and uncultivated TM7 microbes from the human mouth. Proc Natl Acad Sci U S A 104: 1889–11894. 17620602 10.1073/pnas.0704662104 PMC1924555

[12] Swan BK, Martinez-Garcia M, Preston CM, Sczyrba A, Woyke T, et al (2011) Potential for chemolithoautotrophy among ubiquitous bacteria lineages in the dark ocean. Science 333: 1296–1300. 21885783 10.1126/science.1203690

[13] Lage JM, Leamon JH, Pejovic T, Hamann S, Lacey M, et al (2003) Whole genome analysis of genetic alterations in small DNA samples using hyperbranched strand displacement amplification and array-CGH. Genome Res 13: 294–307. 12566408 10.1101/gr.377203 PMC420367

[14] Pinard R, de Winter A, Sarkis G, Gerstein M, Tartaro K, et al (2006) Assessment of whole genome amplification-induced bias through high- throughput, massively parallel whole genome sequencing. BMC Genomics 7: 216. 16928277 10.1186/1471-2164-7-216 PMC1560136

[15] Lasken RS, Stockwell TB (2007) Mechanism of chimera formation during the multiple displacement amplification reaction. BMC Biotechnol 7: 19. 17430586 10.1186/1472-6750-7-19 PMC1855051

[16] Zhang K, Martiny AC, Reppas NB, Barry KW, Malek J, et al (2006) Sequencing genomes from single cells by polymerase cloning. Nat Biotechnol 24: 680–686. 16732271 10.1038/nbt1214

[17] Hutchison CA, Venter JC (2006) Single-cell genomics. Nat Biotechnol 24: 657–658. 16763593 10.1038/nbt0606-657

[18] Spits C, Le Caignec C, De Rycke M, Van Haute L, Van Steirteghem A, et al (2006) Optimization and evaluation of single-cell whole-genome multiple displacement amplification. Hum Mutat 27: 496–503. 16619243 10.1002/humu.20324

[19] Pan X, Urban AE, Palejev D, Schulz V, Grubert F, et al (2008) A procedure for highly specific, sensitive, and unbiased whole-genome amplification. Proc Natl Acad Sci U S A 105: 15499–15504. 18832167 10.1073/pnas.0808028105 PMC2563063

[20] Bredel M, Bredel C, Juric D, Kim Y, Vogel H, et al (2005) Amplification of whole tumor genomes and gene-by-gene mapping of genomic aberrations from limited sources of fresh-frozen and paraffin-embedded DNA. J Mol Diagn 7: 171–182. 15858140 10.1016/S1525-1578(10)60543-0 PMC1867518

[21] Rodrigue S, Malmstrom RR, Berlin AM, Birren BW, Henn MR, et al (2009) Whole genome amplification and de novo assembly of single bacterial cells. PLoS ONE. 4: e6864. 10.1371/journal.pone.0006864 PMC2731171 19724646

[22] RocheDiagnostic (2011): Rapid Library Preparation Method Manual GS FLX+ Series - XL+, May 2011.

[23] Meyer M, Briggs AW, Maricic T, Höber B, Höffner B, et al (2008) From micrograms to picograms: quantitative PCR reduces the material demands of high-throughput sequencing. Nucleic Acids Res 36: e5. 18084031 10.1093/nar/gkm1095 PMC2248761

[24] Zheng Z, Advani A, Melefors O, Glavas S, Nordström H, et al (2010) Titration-free massively parallel pyrosequencing using trace amounts of starting material. Nucleic Acids Res 38: e137. 20435675 10.1093/nar/gkq332 PMC2910068

[25] Huang J, Zheng Z, Andersson AF, Engstrand L, Ye W (2011) Rapid screening of complex DNA samples by single-molecule amplification and sequencing. PloS ONE 6: e19723. 21625543 10.1371/journal.pone.0019723 PMC3098247

[26] Zheng Z, Advani A, Melefors O, Glavas S, Nordstrom H, et al (2011) Titration-free 454 sequencing using y adapters. Nat Protoc 6: 1367–1376. 21886102 10.1038/nprot.2011.369

[27] White R, Blainey P, Fan H C, Quake S (2009) Digital PCR provides sensitive and absolute calibration for high throughput sequencing. BMC Genomics 10: 116. 19298667 10.1186/1471-2164-10-116 PMC2667538

[28] Buehler B, Hogrefe HH, Scott G, Ravi H, Pabón-Peña C, et al (2010) Rapid quantification of DNA libraries for next-generation sequencing. Methods 50: S15–S18. 20215015 10.1016/j.ymeth.2010.01.004

[29] Lennon N, Lintner R, Anderson S, Alvarez P, Barry A, et al (2010) A scalable, fully automated process for construction of sequence-ready barcoded libraries for 454. Genome Biol 11: R15. 20137071 10.1186/gb-2010-11-2-r15 PMC2872875

[30] Buh Gasparic M, Tengs T, La Paz JLL, Holst-Jensen A, Pla M, et al (2010) Comparison of nine different real-time PCR chemistries for qualitative and quantitative applications in GMO detection. Anal Bioanal Chem 396: 2023–2029. 20087729 10.1007/s00216-009-3418-0

[31] Woyke T, Sczyrba A, Lee J, Rinke C, Tighe D, et al (2011) Decontamination of MDA reagents for single cell whole genome amplification. PLoS ONE 6: e26161. 22028825 10.1371/journal.pone.0026161 PMC3197606

[32] Dupont CL, Rusch DB, Yooseph S, Lombardo MJ, Richter RA, et al (2011) Genomic insights to SAR86, an abundant and uncultivated marine bacterial lineage. ISME J 6: 1186–1199. 22170421 10.1038/ismej.2011.189 PMC3358033

[33] Stepanauskas R, Sieracki ME (2007) Matching phylogeny and metabolism in the uncultured marine bacteria, one cell at a time. Proc Natl Acad Sci U S A 104: 9052–9057. 17502618 10.1073/pnas.0700496104 PMC1885626

[34] Garcìa-Garcerà M, Gigli E, Sanchez-Quinto F, Ramirez O, Calafell F, et al (2011) Fragmentation of contaminant and endogenous DNA in ancient samples determined by shotgun sequencing; prospects for human palaeogenomics. PLoS ONE 6: e24161. 21904610 10.1371/journal.pone.0024161 PMC3164143

[35] Danhorn T, Young CR, DeLong EF (2012) Comparison of large-insert, small-insert and pyrosequencing libraries for metagenomic analysis. ISME J 6: 2056–2066. 22534608 10.1038/ismej.2012.35 PMC3475381

[36] Liu JM, Livny J, Lawrence MS, Kimball MD, Waldor MK, et al (2009) Experimental discovery of sRNAs in vibrio cholerae by direct cloning, 5S/tRNA depletion and parallel sequencing. Nucleic Acids Res 37: e46. 19223322 10.1093/nar/gkp080 PMC2665243

[37] Wicker T, Taudien S, Houben A, Keller B, Graner A, et al (2009) A whole-genome snapshot of 454 sequences exposes the composition of the barley genome and provides evidence for parallel evolution of genome size in wheat and barley. Plant J 59: 712–722. 19453446 10.1111/j.1365-313X.2009.03911.x

[38] Hroudova M, Vojta P, Strnad H, Krejcik Z, Ridl J, et al (2012) Diversity, phylogeny and expression patterns of pou and six homeodomain transcription factors in hydrozoan jellyfish craspedacusta sowerbyi. PLoS ONE 7: e36420. 22558464 10.1371/journal.pone.0036420 PMC3340352

[39] Jiang Z, Zhang X, Deka R, Jin L (2005) Genome amplification of single sperm using multiple displacement amplification. Nucleic Acids Res 33: e91. 15942023 10.1093/nar/gni089 PMC1143700

[40] Le Caignec C, Spits C, Sermon K, De Rycke M, Thienpont B, et al (2006) Single-cell chromosomal imbalances detection by array CGH. Nucleic Acids Res 34: e68. 16698960 10.1093/nar/gkl336 PMC3303179

[41] Iwamoto K, Bundo M, Ueda J, Nakano Y, Ukai W, et al (2007) Detection of chromosomal structural alterations in single cells by SNP arrays: A systematic survey of amplification bias and optimized workflow. PLoS ONE 2: e1306. 18074030 10.1371/journal.pone.0001306 PMC2111048

[42] Willner D, Thurber RVV, Rohwer F (2009) Metagenomic signatures of 86 microbial and viral metagenomes. Environ Microbiol 11: 1752–1766. 19302541 10.1111/j.1462-2920.2009.01901.x

[43] Ausubel M, Brent R, Kingston RE, Moore DD, Seidman JG, et al.. (1989): Current protocols in molecular biology. New York: Greene Publishing Associates and Wiley-Interscience. 600 p.

[44] Altschul SF, Gish W, Miller W, Myers EW, Lipman DJ (1990) Basic local alignment search tool. J Mol Biol 215: 403–410. 2231712 10.1016/S0022-2836(05)80360-2

[45] Pages H, Aboyoun P, Gentleman R, DebRoy S (2012) Biostrings: String objects representing biological sequences, and matching algorithms. R package.

[46] R Core Development Team: R (2011) A Language and Environment for Statistical Computing. Vienna: R Foundation for Statistical Computing. 1731 p.

[47] Morgan M, Anders S, Lawrence M, Aboyoun P, Pagès H, et al (2009) Shortread: a bioconductor package for input, quality assessment and exploration of high-throughput sequence data. Bioinformatics 25: 2607–2608. 19654119 10.1093/bioinformatics/btp450 PMC2752612

[48] Hausser J, Strimmer K (2012) entropy: Entropy and Mutual Information Estimation. R package.

[49] Ning Z, Cox AJ, Mullikin JC (2001) SSAHA: a fast search method for large DNA databases. Genome Res 11: 1725–1729. 11591649 10.1101/gr.194201 PMC311141

[50] Li H, Handsaker B, Wysoker A, Fennell T, Ruan J, et al (2009) The sequence Alignment/Map format and SAMtools. Bioinformatics 25: 2078–2079. 19505943 10.1093/bioinformatics/btp352 PMC2723002

[51] Sarkar D, Gentleman R, Lawrence M, Yao Z (2013) chipseq: A package for analyzing chipseq data. R package.

[52] Xiao Y (2012) Cramer-von Mises Two Sample Tests. R package.

[53] Li W, Godzik A (2006) Cd-hit: a fast program for clustering and comparing large sets of protein or nucleotide sequences. Bioinformatics 22: 1658–1659. 16731699 10.1093/bioinformatics/btl158

[54] Oksanen J, Blanchet FG, Kindt R, Legendre P, Minchin PR, et al.. (2013) Community Ecology Package. R package.

[55] Suzuki R., Shimodaira H (2013) Hierarchical Clustering with P-Values via Multiscale Bootstrap Resampling. R package.

