# Correction: Direct Sequencing from the Minimal Number of DNA Molecules Needed to Fill a 454 Picotiterplate

**DOI:** 10.1371/journal.pone.0102719

**Published:** 2014-07-08

**Authors:** The PLOS ONE Staff

## Notice of Republication

This article was republished on June 17, 2014, to correct an error in the title. The publisher apologizes for the error. In addition, a typographical error was corrected in the Abstract. Please download this article again to view the correct version. The originally published, uncorrected article and the republished, corrected article are provided here for reference.

## Supporting Information

File S1 Originally published, uncorrected article.
(PDF)

File S2 Republished, corrected article.
(PDF)
