# Supplementary material for: Direct Sequencing from the Minimal Number of DNA Molecules Needed to Fill a 454 Picotiterplate
Source: PLoS One. 2014 Jun 2;9(6):e97379. doi: 10.1371/journal.pone.0097379 (PMC4041646; doi:10.1371/journal.pone.0097379)
Supplement: Figure S1 — Steps in standard Rapid Library Preparation GS FLX+ Series – XL+ (May 2011) compared with optimized protocol used in this work. The main changes include sonication for DNA fragmentation (not nebulization), small fragments were removed exclusively with AMPure beads, and alternative chemicals were used for library preparation. The library quality was checked with a test PCR with emPCR primers (instead of using Agilent analyzer) and quantified with qPCR instead of a fluorometer. (PDF) [file pone.0097379.s001.pdf]

	Standard Rapid Library Preparation Method Manual, FLX+ Roche	Optimized protocol for direct sequencing of limited samples
Imput DNA amount	1 µg	Even less than 50 pg
DNA Fragmentation	Nebulization	Sonication
Small Fragment removal	Qiagen MinElute PCR Purification kit	AMPure Beads
Fragment End Repair	Fragment End Repair mix, Roche	Fragment End Repair mix, any provider
Adaptor-Ligation	Roche Adaptors / MID Adaptors	Y Adaptors with MIDs designed by Zheng et al. 2010
Small Fragment Removal	AMPure beads	AMPure beads
Library Quality Control	Agilent Bioanalyzer High Sensitivity DNA chip	PCR with emPCR primers, loading PCR product on agarose gel
Quantification method	Fluorometer	qPCR designed by Zheng et al. 2010
Detection limit	1 pg / µL	zeptograms
If not sufficient amount of library is obtained	Repeat the library preparation from the beginning - perform new DNA extraction	Amplify the library with few cycles with emPCR primers to reach the minimal required concentration for sequencing and continue

emPCR and sequencing, standard Roche protocol
