# Supplementary material for: Direct Sequencing from the Minimal Number of DNA Molecules Needed to Fill a 454 Picotiterplate
Source: PLoS One. 2014 Jun 2;9(6):e97379. doi: 10.1371/journal.pone.0097379 (PMC4041646; doi:10.1371/journal.pone.0097379)

A

Mean read length

		Total processed reads	E. coli mapped reads	Unassigned reads
Multiple displacement amplification	Run 1	277.00	267.60	276.10
	Run 2	159.10	237.20	158.20
Direct sequencing	Run 1	230.00	240.20	177.60
	Run 2	139.60	143.60	122.10

Median read length

		Total processed reads	E. coli mapped reads	Unassigned reads
Multiple displacement amplification	Run 1	245.00	225.00	244.00
	Run 2	131.00	169.00	131.00
Direct sequencing	Run 1	203.00	214.00	159.00
	Run 2	119.00	123.00	108.00

GC content

		Total processed reads	E. coli mapped reads	Unassigned reads
Multiple displacement amplification	Run 1	46.05	48.71	46.02
	Run 2	46.15	44.96	46.19
Direct sequencing	Run 1	48.94	48.95	49.57
	Run 2	48.54	48.89	49.36

Read quality

		Total processed reads	E. coli mapped reads	Unassigned reads
Multiple displacement amplification	Run 1	35.92	35.45	35.94
	Run 2	30.84	30.69	30.84
Direct sequencing	Run 1	36.18	36.21	35.90
	Run 2	31.12	31.10	31.19

Read complexity

		Total processed reads	E. coli mapped reads	Unassigned reads
Multiple displacement amplification	Run 1	3.85	3.83	3.85
	Run 2	3.73	3.77	3.73
Direct sequencing	Run 1	3.83	3.85	3.72
	Run 2	3.70	3.73	3.62

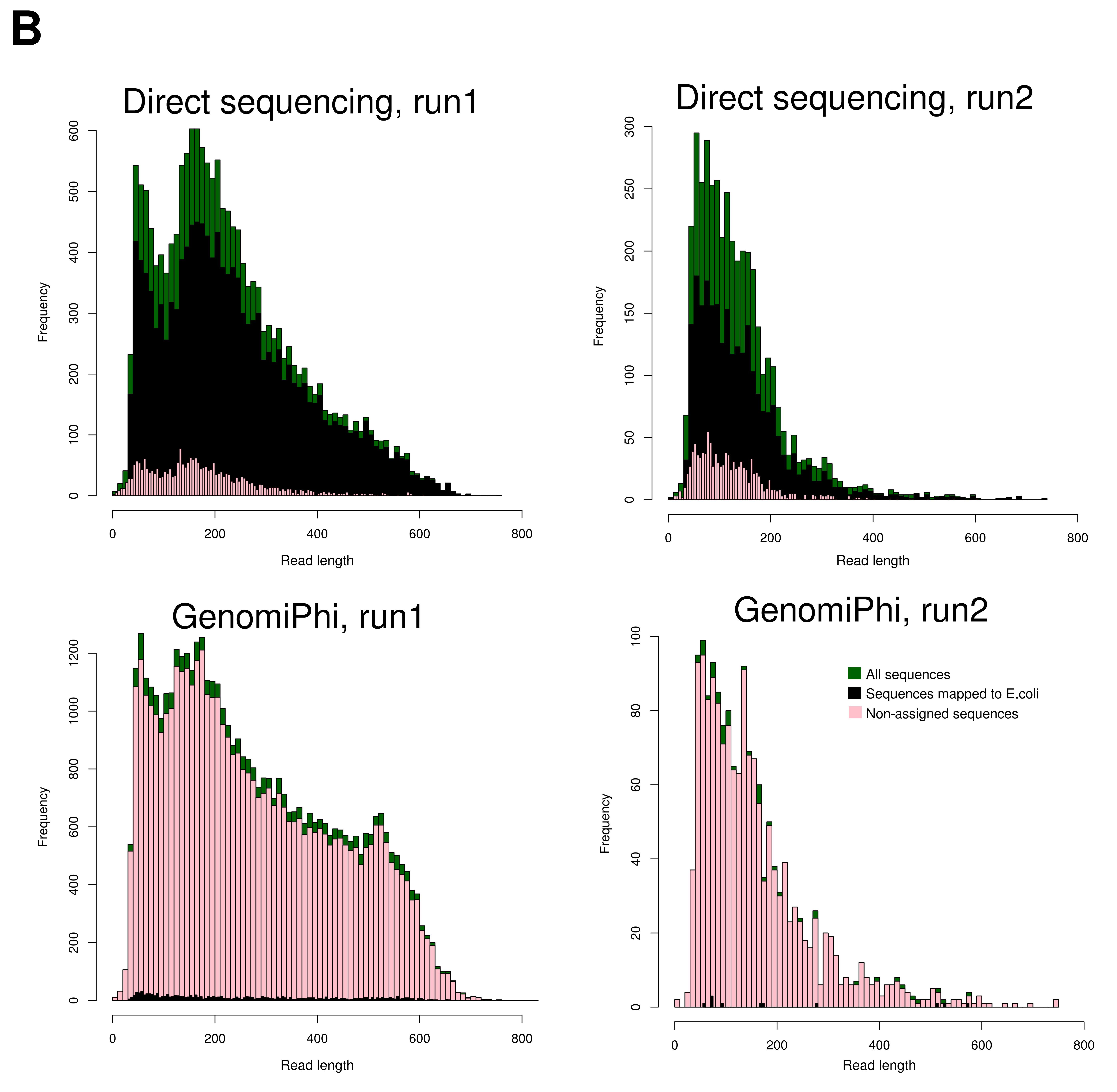

Supplement: Figure S2 — Panel A: Comparison of read length, read complexity, sequence quality and GC content among total reads, E. coli mapped reads and unassigned reads of both approaches (MDA and DS) and both runs. The results in the Table indicate that run 2 performed worse than run 1, but both runs confirmed the same results. The difference between MDA and DS datasets can be observed here only by lower GC content in MDAsample. Panel B: Distribution of read lengths. Distribution of length of total processed reads, reads mapped to E. coli and unclassified reads are compared between both runs of MDA and DS. No differences were found. (PDF) [file pone.0097379.s002.pdf]
