# Supplementary figures and images for: Direct Sequencing from the Minimal Number of DNA Molecules Needed to Fill a 454 Picotiterplate
Source: PLoS One. 2014 Jun 2;9(6):e97379. doi: 10.1371/journal.pone.0097379 (PMC4041646; doi:10.1371/journal.pone.0097379)

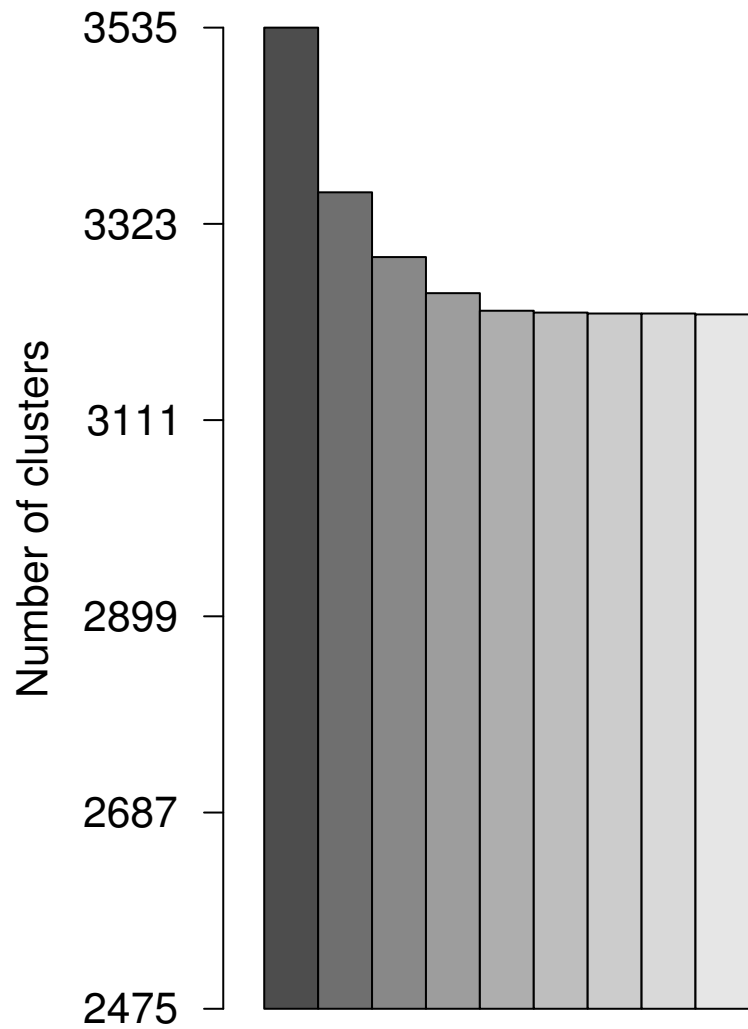

Direct sequencing

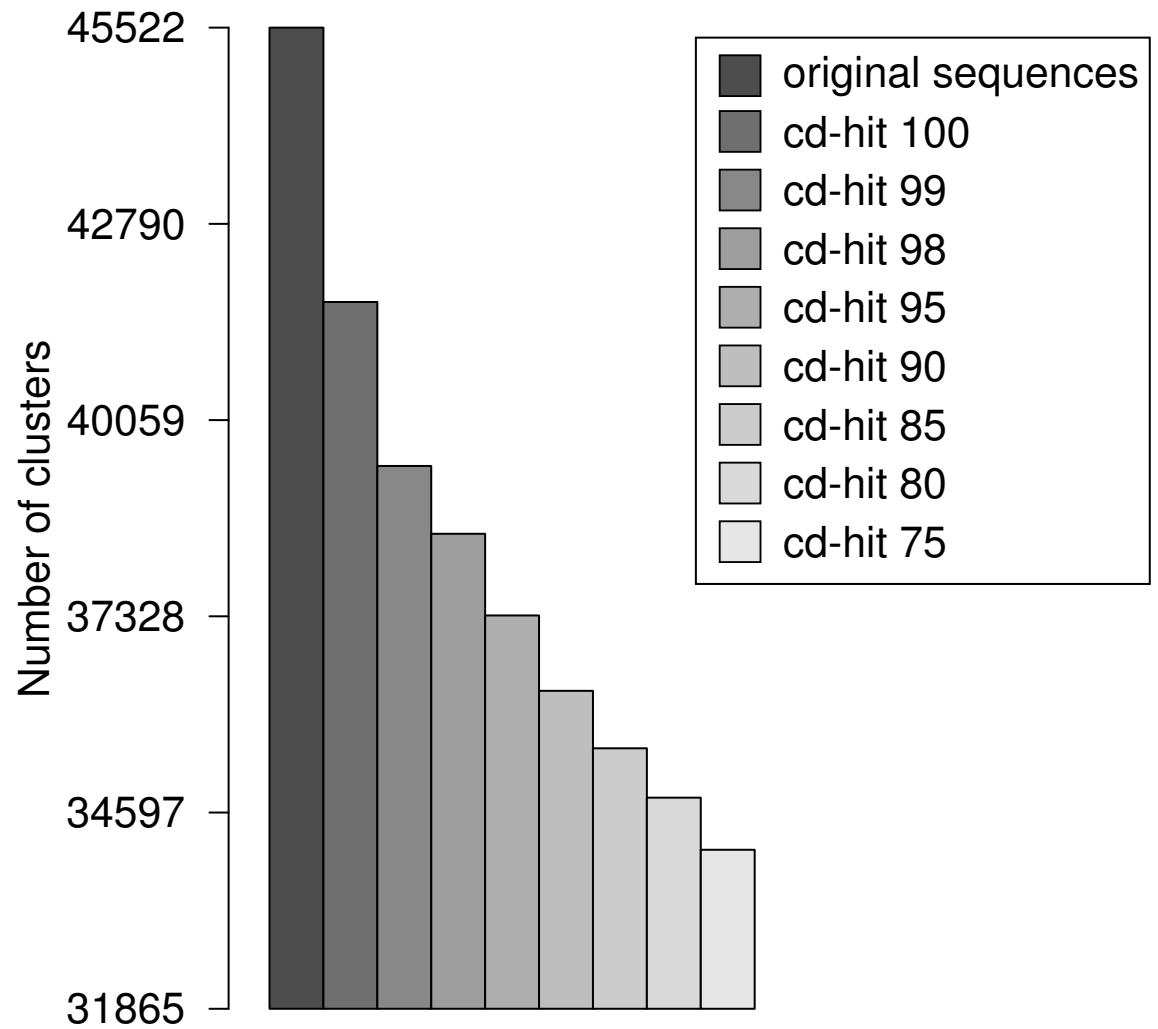

Multiple displacement  
amplification

Supplement: Figure S3 — Clustering of unclassified reads with CD-HIT. The sequences that were not assigned to any species were clustered on different sequence identity levels (from 99 to 75%) allowing us to cluster sequences with 80% length of the cluster. This figure shows the decreasing number of clusters (the increasing size of clusters) by decreasing stringency. Both runs (run 1+ run 2) were processed together for each method (MDA and DS). MDAsample was characterized by abrupt clustering, which demonstrates that the MDAsample reads originated by amplification; however, a high number of clusters was still present at 75% identity level, indicating their uniqueness. (PDF) [file pone.0097379.s003.pdf]

# Correspondence Analysis of Hexamer Distribution in Titration-free method VS GenomiPhi

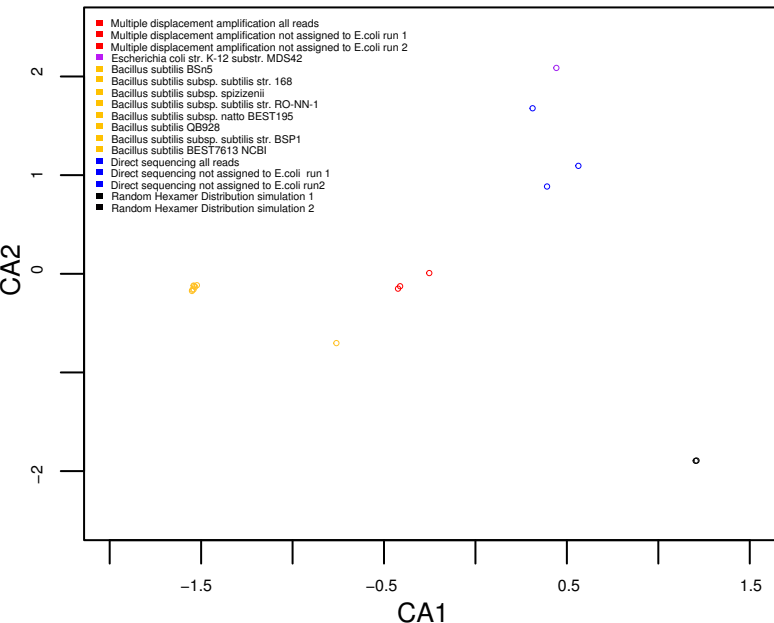

Supplement: Figure S4 — Correspondence analysis of the k-mer relative abundances. Comparison of E. coli, B. subtilis k-mer distributions versus a random k-mer distribution. The taxonomic allocation of the unassigned reads in both methods was obtained by using the eigenvalue coordinates for the k-mer relative abundances for each dataset. (PDF) [file pone.0097379.s004.pdf]

# Correspondence Analysis of Hexamer Distribution in Titration-free method VS GenomiPhi

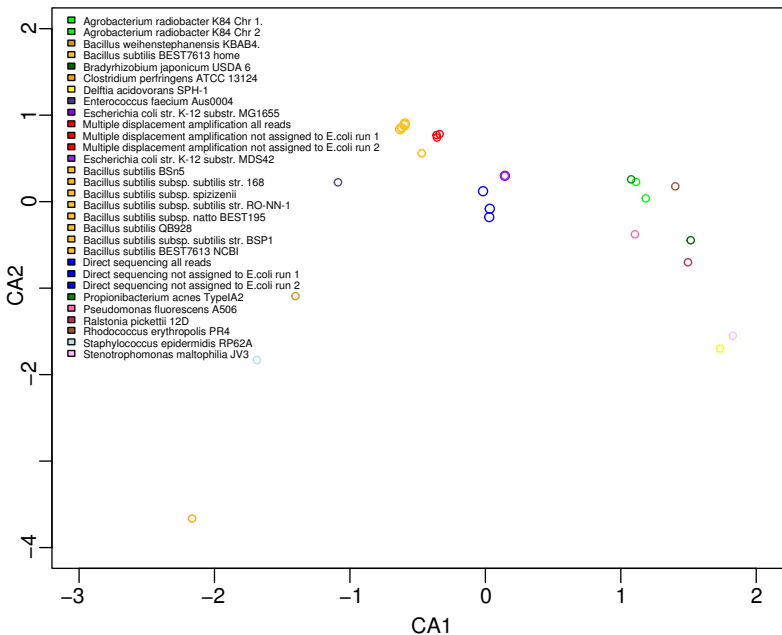

Supplement: Figure S5 — Amplification of the k-mer abundance space spectra on the Figure S4, by including phylogenetically distant genomes on the correspondence analysis. As a result we observe a better aggregation of each methodology dataset to its respective expected phylogenetic source, as observed with other statistical methods. (PDF) [file pone.0097379.s005.pdf]

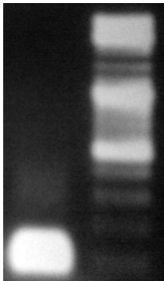

**A-DS-Y3**  
after 2nd purification

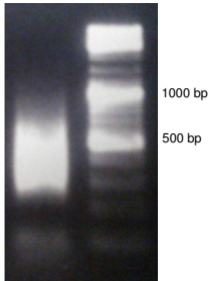

**A-DS-Y3**  
after 4th purification

Supplement: Figure S6 — Quality control of minimal 454 libraries after purification step. This figure shows quality control of libraries DSsample-Y3 and DSsample-Y5 after the 2nd and 4th purification steps. If the concentration of sample DNA fragments is very low, the adaptor:fragment ratio is high and therefore repeated removal of self-ligated adaptors by AMPure beads must be performed. After the first purification, usually only self-ligated adaptors are visible, because they are shorter than the library and therefore amplify better. After each of these purification steps, the amount of self-ligated adaptors is reduced and the library fragments become more visible. (PDF) [file pone.0097379.s006.pdf]

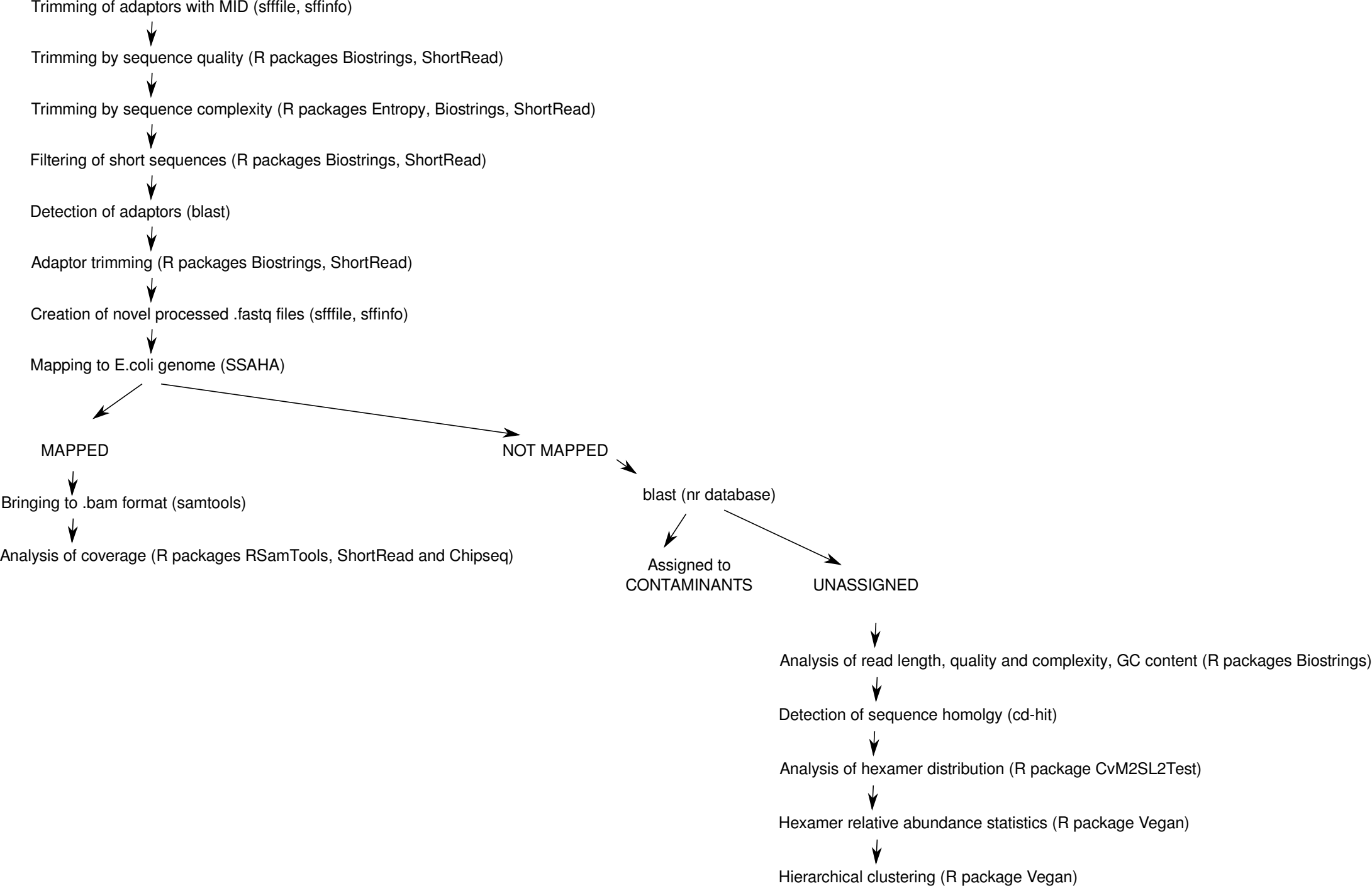

Supplement: Figure S7 — The scheme showing bioinformatics analysis pipeline used in this work. (PDF) [file pone.0097379.s007.pdf]
